# Exosomal miRNA-218–5p derived from low-passage dermal papilla cells modulates hair follicle growth and development

**DOI:** 10.1016/j.ncrna.2026.01.004

**Published:** 2026-01-15

**Authors:** Bohao Zhao, Jiawei Cai, Miaocheng Wang, Jiali Li, Shuyu Yao, Zhiyuan Bao, Yang Chen, Xinsheng Wu

**Affiliations:** a College of Animal Science and Technology, Yangzhou University, 225009, Yangzhou, Jiangsu, China; b Joint International Research Laboratory of Agriculture & Agri-Product Safety, Yangzhou University, 225009, Yangzhou, Jiangsu, China

**Keywords:** Exosome, Hair follicle growth, miR-218–5p

## Abstract

Exosomes are nanoscale particles that participate in multiple biological processes and are secreted by various cell types. Exosomes derived from dermal papilla cells (DPC-Exos) regulate hair follicle (HF) growth and development. In this study, HF development-related genes were significantly expressed in low-passage dermal papilla cells (DPCs). Low-passage DPC-Exos promoted hair follicle stem cell (HFSC) proliferation. After the collection of DPC-Exos, miRNA sequencing identified 36 differentially expressed (DE) miRNAs between low-passage and high-passage DPC-Exos. Among these, miR-218–5p was significantly upregulated in low-passage DPC-Exos and dysregulated HF growth and development-related gene expression in HFSCs. Furthermore, the transport mechanism of exosomal miR-218–5p from DPCs is into HFSCs was verified. RNA sequencing of HFSCs treated with exosomal miR-218–5p identified differentially expressed genes (DEGs). The results indicated involvement of miR-218–5p in signaling pathways related to HF growth and development. Additionally, *in vitro* HF organ culture experiments demonstrated that exosomal miR-218–5p actively promotes hair growth. In summary, low-passage DPC-Exos enhance HFSC proliferation. Exosomal miR-218–5p may serve as a biomarker and therapeutic target for human hair disorders and as a tool to improve wool production in mammals.

## 1 Introduction

Hair follicles (HFs), as skin appendages, are complex miniorgans that support vital activities in mammals, including temperature regulation, body protection, sensory perception, and social behavior [1,2]. HFs exhibit unique regenerative ability in mammals and undergo a periodic cycle of three phases: anagen, catagen, and telogen [3]. Various HF-related cells participate in HF growth, development, and periodic regeneration, including dermal papilla cells (DPCs), hair follicle stem cells (HFSCs), inner root sheath cells (IRSCs), outer root sheath cells (ORSCs), dermal sheath cells (DSCs), and melanocytes [[4], [5], [6], [7]]. HFSCs are mainly located in the convex area adjacent to the hair shaft and hair matrix. They have strong multidirectional differentiation and self-renewal abilities [8]. During skin aging and HF cycle regeneration, HFSCs show imbalanced cytokine signals, and the colony formation ability of HF-located cells decreases [[9], [10], [11]]. DPCs are mesenchymal cells in HFs with potential hair-inducing ability and play a vital role in HF morphogenesis [12]. Proteomic analysis between passage 3 and 9 DPCs showed that early-passage DPCs regulate HF morphogenesis and regeneration through Wnt, TGF-β, and BMP signaling pathways [13]. In mouse embryos at E12.5 days, the dermis exhibited activity that induced HF morphogenesis before dermal papillae formation [14]. Extensive crosstalk between epithelial cells and dermal papillae is involved in HF morphogenesis and regeneration [15].

Extracellular vesicles (EVs) are a heterogeneous group characterized as powerful vehicles of intercellular communication, capable of transferring nucleic acids, lipids, and proteins [16]. Among EVs, cell-derived membrane vesicles include microparticles, microvesicles, and exosomes, which act as key mediators of intercellular communication, regulating multiple biological processes [17]. Exosomes originate from multivesicular bodies through exocytosis and are nanosized particles (40–150 nm) carrying proteins, lipids, mRNA, long non-coding RNA (lncRNA), microRNA (miRNA), and circular RNA (circRNA) [18]. Exosomes participate in HF morphogenesis, growth, and development. For example, exosomes derived from adipose stem cells promoted HF regeneration *in vivo* [19]. Exosomes derived from dermal papilla cells (DPC-Exos) significantly induced anagen and postponed catagen during the HF cycle [20]. Exosomes derived from three-dimensional (3D)-cultured DP cells promoted DPC and ORSC proliferation and influenced HF neogenesis in mice [21]. MiRNAs are secreted through exosomes, which protect them from RNase degradation, indicating that exosomal miRNAs play essential roles in biological processes through intercellular communication [22,23]. Exosomal miRNAs play crucial roles in HF growth and development. For instance, ADSC-Exos carrying miR-122–5p inhibited the TGF-β/SMAD3 axis, promoting HF regeneration in androgenic alopecia [24]. MiR-22–5p was highly expressed in DPC-Exos and regulated HFSC proliferation by targeting *LEF1* [25]. DPC-Exos-derived miR-181a-5p activated HFSC proliferation and promoted HF growth via the Wnt/β-catenin signaling pathway [26]. Enhanced expression of miR-218–5p in HF spheroid-derived exosomes upregulated β-catenin expression and downregulated SFRP2 expression, thereby promoting HF growth by maintaining anagen during the HF cycle [27]. Recent reviews have highlighted the potential of exosomes as therapeutic targets for hair loss, suggested that exosomes have emerged as promising therapeutic agents for alopecia treatment, and recognized DPC-Exos as promising tools for hair regeneration that contain miRNAs regulating HF growth [[28], [29], [30]]. However, the influence of DPC passage on exosomal miRNA profiles and subsequent HF regulation remains unclear, and the specific mechanisms underlying passage-dependent functional differences are not fully elucidated.

In this study, DPC-Exos were isolated and identified from DPCs, and exosomal miRNAs derived from passage one and eight DPC-Exos were investigated. A systematic comparison of miRNA expression profiles between low-passage and high-passage DPC-Exos was performed. Furthermore, the regulatory mechanism of a key miRNA involved in HF growth and development was explored. The study identified a novel miR-218-5p-mediated regulatory network in HFSCs via RNA sequencing and validated the effect of exosomal miR-218–5p in a rabbit HF organ culture system. These findings provide a theoretical reference for human hair disorders and strategies to increase wool yield in mammals.

## 2 Materials and methods

### 2.1 Animals

The dorsal skin and whiskers of rabbits were obtained from six-month-old male Angora rabbits for subsequent research. The rabbits were maintained under controlled environmental conditions. For skin sampling, anesthesia was administered via ear vein injection using Zoteil-50, and sampling sites were disinfected with iodine solution after collection. All animal experimental procedures were approved by the Animal Care and Use Committee of Yangzhou University (Approval No.: 202103358).

### 2.2 Cell isolation, culture, and transfection

DPCs were isolated from HFs of male Angora rabbits and cultured in Mesenchymal Stem Cell Medium (MSCM, Sciencell®, CA, USA, Cat No.7510). HFSCs were isolated from Angora rabbits and cultured in Dulbecco's Modified Eagle Medium: F-12 (DMEM/F-12, Gibco®, USA, Cat No. 10565018), supplemented with 10 % fetal bovine serum (FBS, One ShotTM, Gibco®, USA Cat No. A3160402). All cells were maintained in an incubator at 37 °C with 5 % CO_2_. The cell seeding density was 5 × 10^4^ cells/well for 24-well plates and 1 × 10^4^ cells/well for 96-well plates. MiR-218–5p mimics, miR-218–5p inhibitor, and their respective negative controls (NC) were purchased from Guangzhou RiboBio Co., Ltd. For cell transfection, miR-218–5p mimics (100 nM) and miR-218–5p inhibitor (100 nM) were transfected using Lipofectamine™ 2000 (Invitrogen, CA, USA, Cat. No. 11668019). The cells were harvested 48 h post-transfection for subsequent experiments.

### 2.3 Exosome isolation and identification

To isolate and collect exosomes, the culture medium of DPCs was processed using the Total Exosome Isolation Kit (from cell culture media, Invitrogen®, USA, Cat. No. 4478359) following the manufacturer's protocol. After the isolation of exosomes, total RNA and protein were purified using the Total Exosome RNA and Protein Isolation Kits (Invitrogen®, USA, Cat No. 4478545). The morphology of DPC-Exos was observed by transmission electron microscopy (TEM) using the HT-7700 TEM (Hitachi, Tokyo, Japan). The expression levels of exosome-specific markers were detected through the Wes automated Western blotting system (Protein Simple) following the manufacturer's instructions [31]. Nanoparticle tracking analysis (NTA) was employed to estimate the particle size of DPC-Exos using a ZetaView PMX 110 instrument (Particle Metrix, Meerbusch, Germany). Data were analyzed with ZetaView 8.04.02 software (Particle Metrix, Meerbusch, Germany). For the cell treatment, 10 μg/mL exosomes were used, and the cells were harvested 48 h post-treatment.

### 2.4 Immunofluorescence

Cells were seeded into 24-well plates and cultured for 24 h. Phosphate-buffered saline (PBS, Hyclone, USA, Cat No. SH30256.01) was used for washing, and cells were fixed with 4 % paraformaldehyde. Subsequently, PBS was used again for washing, then cells were permeabilized in 0.3 % Triton X-100 (Beyotime, China, Cat. No. P0096) and blocked with 1 % BSA (Solarbio, China, Cat. No. SW3015). Primary antibodies against alkaline phosphatase (ALPL, Proteintech, China, Cat No. 68719-1-Ig) and proliferating cell nuclear antigen (PCNA, Proteintech, China, Cat No. 60097-1-Ig) were added and incubated overnight at 4 °C. After three times washing with PBS containing 0.1 % Tween-20 (Solarbio, China, Cat. No. ST825), secondary antibodies Cy3-conjugated Goat Anti-Mouse lgG (Proteintech, China, Cat No. SA00009-1) and Fluorescein (FlTc)-conjugated Goat Anti-Mouse lgG (Proteintech, China, Cat No. SA00003-1) were applied for incubation. Finally, the cells were treated with 4′,6-diamidino-2-phenylindole (DAPI, Solarbio, China, Cat. No. C1006) for staining. After three washes with PBS, cells were observed under a fluorescence microscope to capture images.

### 2.5 Co-culture assay

The co-culture system was set up using a Transwell plate equipped with a 0.4 μm polycarbonate filter (Corning Costar, USA, Cat. No. 3412). DPCs were cultured in the upper chamber, and HFSCs were cultured in the lower chamber. Fluorescence in HFSCs was observed after transfection of Cy3-labeled miR-218–5p mimics (Guangzhou RiboBio Co., Ltd.) in DPCs. GW4869 (10 μM; MCE, USA, Cat. No. HY-19363), an inhibitor of EV secretion [32], was used to inhibit exosome release [33]. For the inhibition of miRNA synthesis, the miRNA expression of DPCs was knocked down by transfection with siRNA-Drosha (sequence shown in Table S1). The pretreated DPCs were then co-cultured with HFSCs.

### 2.6 miRNA library construction and sequencing

Exosomes were isolated from the passage 1 (P1) DPCs group (n = 3) and the passage 8 (P8) DPCs group (n = 3) of Angora rabbits. Total RNA was obtained from these DPC-Exos, and the RNA Nano 6000 Assay Kit on the Agilent Bioanalyzer 2100 system (Agilent Technologies, CA, USA) was used for RNA integrity assessment. For miRNA library construction and sequencing, total RNA from six samples was used as input material separately. Libraries were prepared with the NEBNext® Multiplex Small RNA Library Prep Set for Illumina® (San Diego, CA, USA). Library quality was evaluated using the Agilent Bioanalyzer 2100 system. Following cluster generation, the Illumina SE50 platform was used for sequencing, which produced 50 bp single-end reads. After removal of 5′ adapter contaminants, reads lacking 3′ adapters, poly-N, homopolymers (poly A/T/G/C), insert tags, and low-quality reads, raw data were acquired. After quality control, reference genome mapping of small RNA tags was performed using bowtie2 [34]. The quality control and mapping rate data are listed in Table S2. The reported *Oryctolagus cuniculus* miRNAs in the miRBase 20.0 database were used for aligning known miRNAs, and novel miRNAs were predicted by the mirdeep2 tool [35], which analyzes secondary structures and hairpin motifs of miRNA precursors. MiRNA expression levels were quantified by transcripts per million (TPM) [36]. The differential expression levels of miRNAs between groups were analyzed using the DESeq R package (version 3.0.3) [37], with a threshold of |log_2_ (fold change)| > 1 and *P*-value <0.05 defining significant differences. Raw reads of miRNA sequencing were deposited in the Short Read Archive (SRA) of the National Center for Biotechnology Information (NCBI) under accession number PRJNA839153.

### 2.7 RNA sequencing

HFSCs were incubated for 48 h with two groups: the DPC-Exo^miR^^−218−5p^ group (treated with DPC-Exos overexpressing miR-218–5p mimics, n = 3) and the control group (treated with DPC-Exos transfected with miR-218–5p mimic NC, n = 3). Total RNA was collected from HFSCs transfected with the miR-218–5p mimics and miR-218–5p mimics NC for 48 h. The Agilent 2100 Bioanalyzer (Agilent Technologies, USA) and a NanoDrop 2000 spectrophotometer (Thermo Scientific, USA) were used for RNA quality assessment. Library construction for each group was conducted using the Hieff NGS Ultima Dual-mode mRNA Library Prep Kit for Illumina (Yeasen Biotechnology Co., Ltd., Cat No. 12310 ES). The Illumina NovaSeq PE150 platform was used for RNA sequencing, following the manufacturer's protocols. After quality control, genome alignment of clean reads to the rabbit reference genome (*OryCun2.0*) was carried out using HISAT2 tools [38]. The quality control and mapping rate data are listed in Table S3. Gene expression levels were estimated with fragments per kilobase of transcript per million mapped fragments (FPKM). Differential expression analysis was performed with DESeq2 [39], using the criteria |log_2_ (fold change)| > 1 and False Discovery Rate (FDR) < 0.01 to identify differentially expressed (DE) genes. RNA sequencing raw reads were deposited in the Short Read Archive (SRA) under accession number PRJNA1141892.

### 2.8 Target gene prediction and Gene Ontology and Kyoto Encyclopedia of Genes and Genomes enrichment analyses

The prediction of miRNA target genes was conducted using miRanda software (version 3.3a) with the following parameters: sc 140; -en −10; -scale 4; -strict [40]. With the clusterProfiler (version 3.8.1) R package, candidate target genes of DE miRNAs and DEGs were subjected to Gene Ontology (GO) enrichment analysis. Both the KOBAS software [41] and the clusterProfiler (3.8.1) R package were employed for Kyoto Encyclopedia of Genes and Genomes (KEGG) pathway analysis for targeted genes and DEGs.

### 2.9 Wes automated western blotting system and western blotting

Exosomes or cell lysates were collected using the RIPA Lysis Buffer (PPLYGEN, Beijing, China, Cat No. P0013B) following the manufacturer's instructions. Protein concentrations were quantified by the Enhanced BCA Protein Kit (Beyotime, China, Cat No. P0012). For exosome identification, protein samples were analyzed via the Wes automated Western blotting system (Protein Simple) following the manufacturer's instructions [31]. Exosome protein lysates were probed with anti-TSG101 mouse monoclonal antibody (Proteintech, China, Cat No. 67381-1-Ig), anti-CD9 mouse polyclonal antibody (Proteintech, China, Cat No. 20597-1-AP), and anti-Calnexin monoclonal antibody (Proteintech, China, Cat No. 66903-1-Ig). Western blot analysis of HFSCs was performed according to a standard protocol. Anti-SFRP2 rabbit polyclonal antibody (Proteintech Biotech, Cat No. 12189-1-AP), anti-β-catenin polyclonal antibody (Proteintech, China, Cat No. 51067-2-AP), and anti-GAPDH mouse monoclonal antibody (Proteintech, China, Cat No. 60004-1-Ig) were the primary antibodies.

### 2.10 qPCR with reverse transcription (RT-qPCR)

The RNAsimple Total RNA Kit (Tiangen, China, Cat No. DP419) was used for total RNA collection from exosomes, HFs, and cells. For mRNA analysis, cDNA was obtained with the HiScript II Q Select RT SuperMix (Vazyme, China, Cat No. R233-01). According to the manufacturer's instructions, the ChamQ SYBR qPCR Master Mix (Vazyme, China, Cat No. Q311-02) on a QuantStudio® 5 instrument (Applied Biosystems) was used for reverse transcription-quantitative PCR (RT-qPCR). For miRNA expression detection, cDNA was synthesized using the miRcute Plus miRNA First-Strand cDNA Synthesis Kit (Tiangen, China, Cat No. KR211). The miRcute miRNA qPCR Detection Kit (SYBR Green, Cat No. FP411) was used for RT-qPCR detection. The 2^−ΔΔCt^ method was conducted to estimate relative expression levels of mRNAs and miRNAs [42], with GAPDH or U6 served as housekeeping genes. The primer sequences are provided in Table S4.

### 2.11 Cell proliferation and apoptosis assay

The Cell Counting Kit-8 (CCK-8, Vazyme, China, Cat No. A311) was used for cell proliferation assessment following the manufacturer's protocol. The 450 nm optical density (OD) values were measured at 72 h using an Infinite M200 pro (Tecan, Switzerland). The Annexin V-FITC Apoptosis Detection Kit (Vazyme, China, Cat No. A214) was employed, and apoptosis rates were analyzed via flow cytometry using a FACSAria SORP instrument (Becton Dickinson, USA). Data were processed with FlowJo V10 software (FlowJo, OH, USA) to quantify apoptosis rates.

### 2.12 The *in vitro* organ culture of the hair follicles

The whisker tissues of Angora rabbits were used for HF isolation, and an *in vitro* HF organ culture system was established following a previously described protocol [43]. HF organs were randomly divided into two groups: the DPC-Exo miR^−218−5p^-treated group and the control group. The growth status of HFs and hair shaft length were examined using a light microscope (Olympus BX43, Tokyo, Japan). HFs were collected for subsequent experiments on day 4.

### 2.13 Statistical analysis

SPSS 22.0 software (SPSS, USA) was used for analysis of relative expression levels of genes and miRNAs, OD values, apoptosis rates, and hair shaft lengths. An unpaired two-tailed *t*-test was used for two-group comparisons, and one-way ANOVA with Tukey post hoc test was used for multiple-group comparisons. The results are presented as mean ± standard error (SE) with at least three biological replicates per analysis. A *P*-value <0.05 was considered statistically significant.

## 3 Results

### 3.1 Characterization of DPC-Exos

**Fig. 1:**
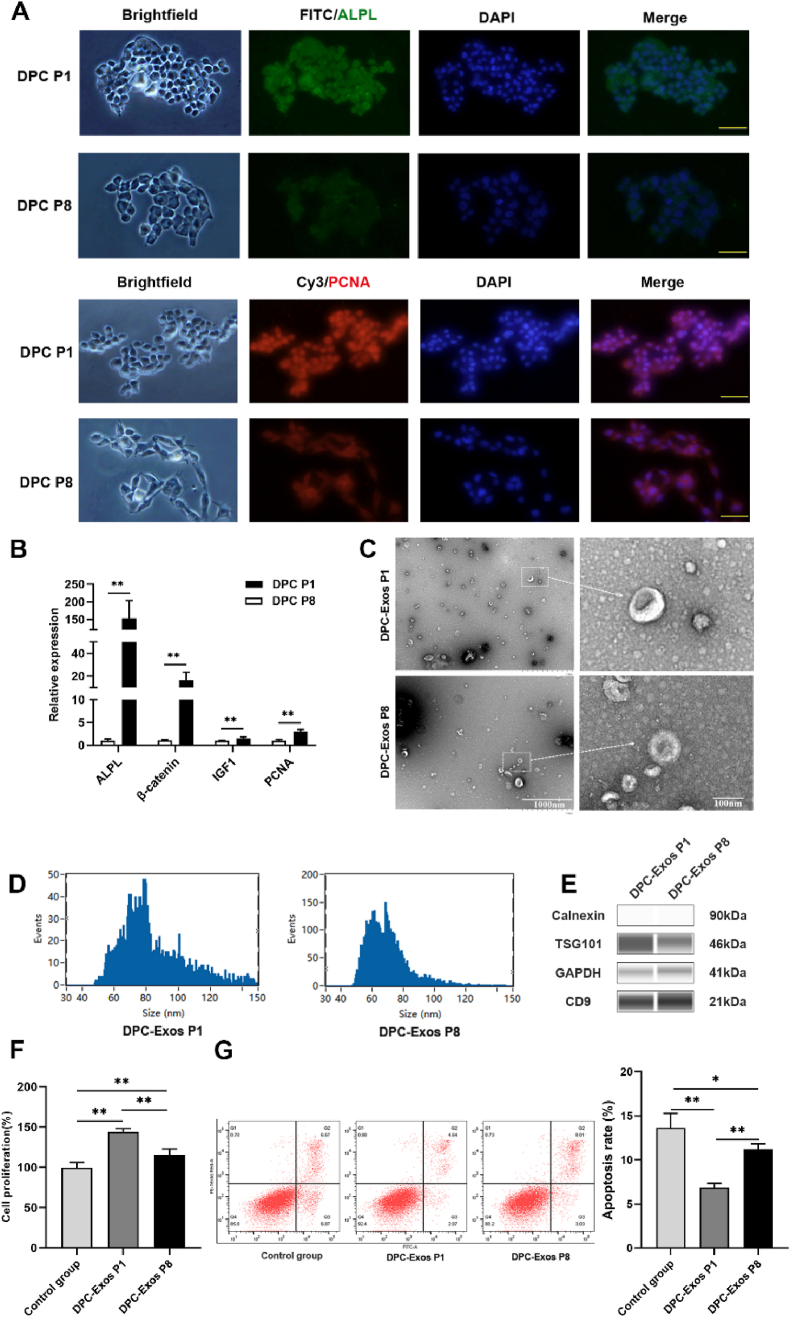
**Exosomes derived from low-passage DPCs regulated HFSC proliferation.** (A) Indirect immunofluorescence showing ALPL and PCNA expression in low-passage (P1) and high-passage (P8) DPCs (scale bar = 50 μm). (B) RT-qPCR analysis of HF development-related gene expression in P1 and P8 DPCs (unpaired two-tailed *t*-test, n = 3). (C) TEM images of exosomes from P1 DPCs (DPC-Exos P1) and P8 DPCs (DPC-Exos P8). (D) NTA measurement of particle size of DPC-Exos P1 and DPC-Exos P8. (E) Western blot detection of exosome-specific proteins in DPC-Exos P1 and DPC-Exos P8. (F) CCK-8 assay evaluating HFSC proliferation after treatment with DPC-Exos from P1 and P8 (one-way ANOVA, n = 5). (G) Flow cytometry analysis of HFSC apoptosis after treatment with DPC-Exos from P1 and P8 (one-way ANOVA, n = 3). ∗*P* < 0.05, ∗∗*P* < 0.01.

The low- and high-passage Angora rabbit DPCs were collected. Immunofluorescence revealed that low-passage (1st passage, P1) DPCs had higher expression of alkaline phosphatase (ALPL) and proliferating cell nuclear antigen (PCNA) than high-passage (8th passage, P8) DPCs (Fig. 1A). The expression levels of HF development-related genes, including *ALPL*, *β-catenin*, *IGF-1*, and *PCNA*, were higher in P1 DPCs than in P8 DPCs (Fig. 1B). Exosomes were isolated from P1 and P8 DPCs. TEM analysis of DPC-Exos morphology showed cup- or round-shaped forms (Fig. 1C). NTA revealed that the mean diameter of P1 DPC-Exos was 83.45 ± 19.94 nm, while P8 DPC-Exos measured 69.85 ± 13.66 nm (Fig. 1D). This size difference may be associated with the exosome yield, which is influenced by the passage number of parental cells. Western blots confirmed expression of exosome-specific surface markers TSG101 and CD9 in both P1 and P8 DPC-Exos (Fig. 1E). After transfection of DPC-Exos into HFSCs, P1 DPC-Exos significantly increased cell proliferation compared with P8 DPC-Exos or untreated control (*P* < 0.01, Fig. 1F). P1 DPC-Exos significantly inhibited HFSC apoptosis compared to P8 DPC-Exos or untreated control (*P* < 0.01, Fig. 1G). These results indicated that low-passage DPC-Exos activated HFSC proliferation.

### 3.2 Comparison of miRNA expression profiles between low-passage and high-passage DPC-Exos

**Fig. 2:**
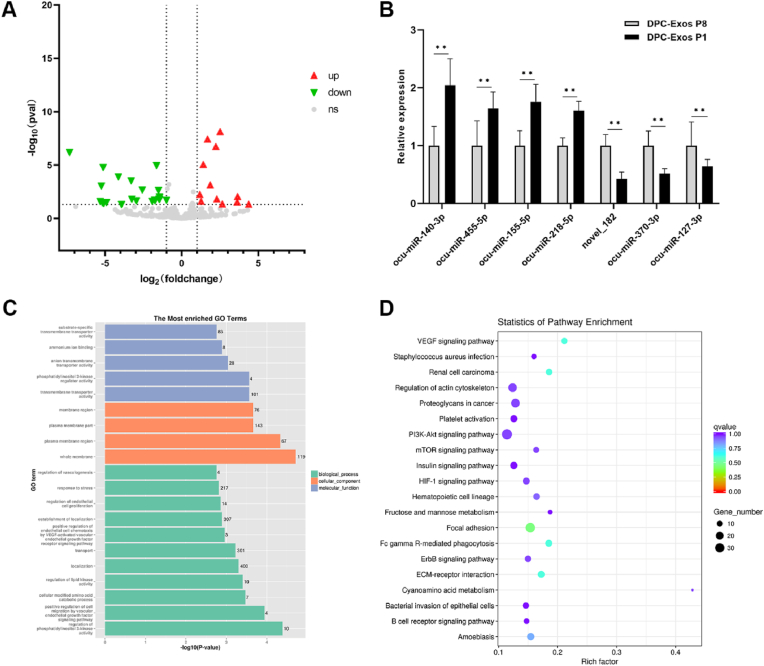
**Differentially expressed miRNAs between DPC-Exos P1 and DPC-Exos P8 identified by miRNA sequencing.** (A) Differential expression analysis of miRNAs between DPC-Exos P1 and DPC-Exos P8. (B) RT-qPCR validation of differential miRNA expression (unpaired two-tailed *t*-test, n = 3). (C) GO enrichment of target genes of DE miRNAs. (D) KEGG enrichment of target genes of DE miRNAs. ∗∗*P* < 0.01.

Illumina HiSeq high-throughput sequencing was used to assess miRNA expression in P1 and P8 DPC-Exos. A total of 63, 239, 926 raw reads were generated, producing 59, 882, 284 clean reads, with a clean read rate exceeding 94 % for each sample. Across six samples, 278 mature miRNAs and 22 novel miRNAs were identified. Differential expression analysis revealed 36 significantly dysregulated miRNAs between P1 and P8 DPC-Exos, including 12 upregulated and 24 downregulated miRNAs (Fig. 2A, Table S5). Seven DE miRNAs were selected for validation. Four miRNAs (miR-140–3p, miR-455–5p, miR-155–5p, and miR-218–5p) were upregulated in the P1 group, while three miRNAs (miR-370–3p, miR-127–3p, and novel_182) were downregulated in the P1 group. These findings were consistent with sequencing results (Fig. 2B). DE miRNA target genes were predicted, followed by GO and KEGG pathway analyses to explore molecular functions. GO enrichment analysis identified significantly enriched terms (Fig. 2C), including whole membrane (GO:0098,805), regulation of phosphatidylinositol 3-kinase activity (GO:0043,551), transmembrane transporter activity (GO:0038,089), and plasma membrane region (GO:0098,590). Terms related to HF development were also identified, including hair follicle morphogenesis (GO:0031,069), hair follicle maturation (GO:0048,820), and regulation of hair follicle development (GO:0051,797). KEGG signaling pathway analysis revealed enrichment in focal adhesion, Fc gamma R-mediated phagocytosis, and VEGF signaling (Fig. 2D). HF development-related pathways, including HIF-1, PI3K-Akt, Hedgehog, and Wnt signaling pathways, were enriched among DE miRNA target genes.

### 3.3 DPC-exos containing miR-218–5p can be transferred from DPCs to HFSCs

**Fig. 3:**
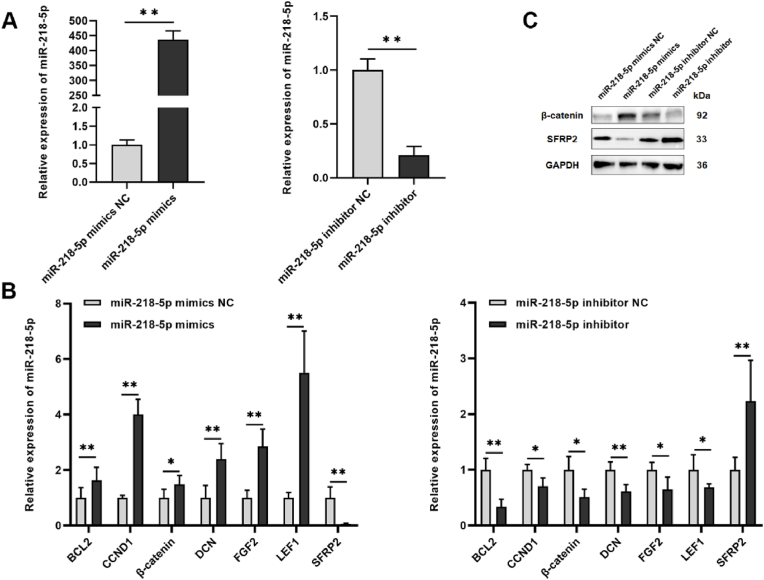
**MiR-218-5p regulated HF growth- and development-related gene expression in HFSCs.** (A) MiR-218–5p expression levels in HFSCs after transfection with miR-218–5p mimics or the inhibitor (unpaired two-tailed *t*-test, n = 3). (B) Expression of HF development-related genes in HFSCs is regulated by miR-218–5p. (C) β-Catenin and SFRP2 protein expression in HFSCs after treatment with miR-218–5p mimics or inhibitor (unpaired two-tailed *t*-test, n = 3). ∗*P* < 0.05, ∗∗*P* < 0.01.

Based on the DE miRNAs expression profiles in DPC-Exos, miR-218–5p was expressed at significantly higher levels in low-passage DPC-Exos than in high-passage DPC-Exos. Our previous study showed that miR-218–5p positively regulates HF growth and development via the Wnt/β-catenin pathway in rabbits [44]. In this study, overexpression of miR-218–5p significantly increased miR-218–5p expression (*P* < 0.01), while the miR-218–5p inhibitor significantly decreased miR-218–5p expression in HFSCs (*P* < 0.01, Fig. 3A). The mRNA expression levels of HF growth and development-related genes were examined. RT-qPCR results showed that miR-218–5p mimics significantly upregulated expression of *BCL2*, *CCND1*, *β-catenin*, *DCN*, *FGF2*, and *LEF1*, and downregulated *SFRP2* (*P* < 0.01). The miR-218–5p inhibitor significantly downregulated *BCL2*, *CCND1*, *β-catenin*, *DCN*, *FGF2*, and *LEF1*, while upregulating *SFRP2* (*P* < 0.01, Fig. 3B). The miR-218–5p mimics reduced SFRP2 protein levels but increased β-catenin protein levels (Fig. 3C).

**Fig. 4:**
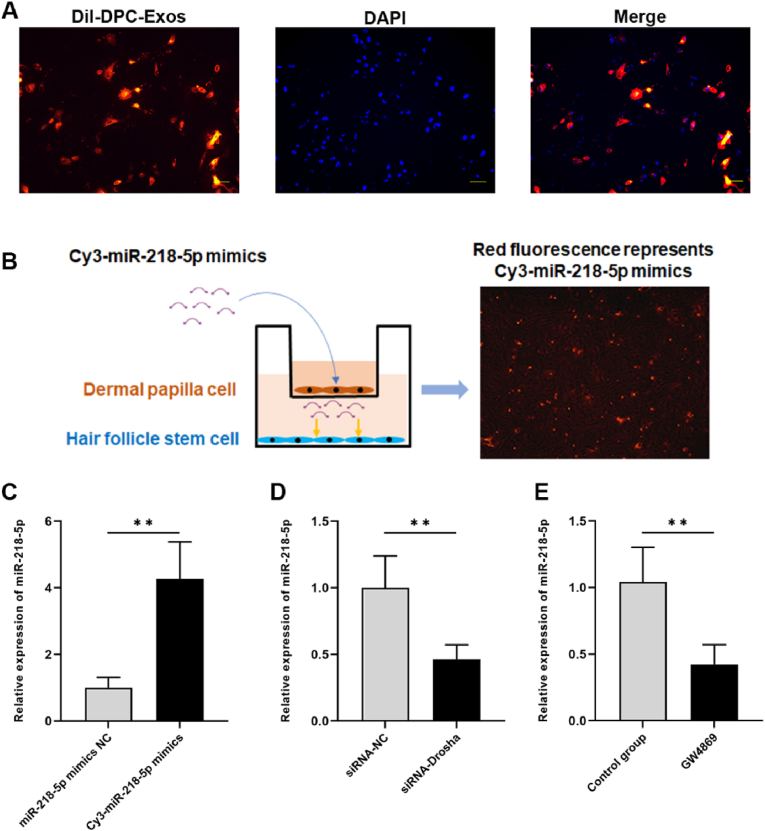
**DPC-Exos transported miR-218-5p from DPCs to HFSCs.** (A) Fluorescence imaging of HFSCs after treatment with DiI-labeled DPC-Exos (scale bar = 50 μm). (B) Schematic of the DPC–HFSC co-culture system with fluorescence showing intercellular transfer of exosomal miR-218–5p. (C) MiR-218–5p expression in HFSCs after transfection with Cy3-miR-218–5p (unpaired two-tailed *t*-test, n = 3). (D) MiR-218–5p expression in HFSCs after DPCs were transfected with siRNA-Drosha (unpaired two-tailed *t*-test, n = 3). (E) MiR-218–5p expression in HFSCs after DPCs were treated with GW4869 (unpaired two-tailed *t*-test, n = 3). ∗∗*P* < 0.01.

To verify the uptake of DPC-Exos in HFSCs, DiI-labeled DPC-Exos (red fluorescence) were added to HFSCs. After culture with DPC-Exos, DiI-labeled exosomes were observed in the cytoplasm of HFSCs under a fluorescence microscope (Fig. 4A). In the co-culture system, HFSCs exhibited red fluorescence from Cy3-miR-218–5p mimics after their transfer from DPCs (Fig. 4B). This suggested that miR-218–5p mimics were delivered from DPCs to HFSCs. Consistent with this, miR-218–5p expression was significantly increased in HFSCs (*P* < 0.01, Fig. 4C). Furthermore, miR-218–5p expression was significantly reduced in HFSCs after DPCs were transfected with siRNA-Drosha (*P* < 0.01, Fig. 4D). Similarly, treating DPCs with GW4869 (an extracellular vesicle secretion inhibitor) reduced miR-218–5p levels in HFSCs (*P* < 0.01, Fig. 4E), suggesting inhibition of exosome production and transfer from DPCs to HFSCs. Hence, miR-218–5p was delivered from DPCs to HFSCs through communication mediated by DPC-Exos.

### 3.4 Identification of DEGs in HFSCs treated with exosomal miR-218–5p

**Fig. 5:**
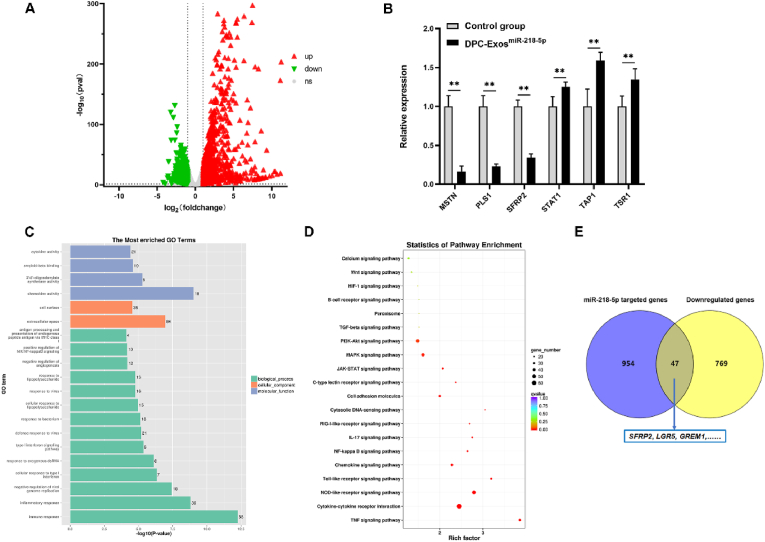
**RNA sequencing identified differentially expressed genes (DEGs) in HFSCs regulated by exosomal miR-218-5p.** (A) Analysis of DEGs by RNA sequencing. (B) RT-qPCR validation of DEG expression levels (unpaired two-tailed *t*-test, n = 3). (C) GO enrichment of DEGs. (D) KEGG enrichment of DEGs. (E) Venn diagram showing overlap between downregulated DEGs and predicted miR-218–5p target genes. ∗∗*P* < 0.01.

DPC-Exo^miR−218−5p^ was applied to HFSCs, and RNA sequencing was performed to screen DEGs between the DPC-Exo^miR−218^^−^^5p^-treated group and the control group. A total of 1947 DEGs were identified, including 1131 upregulated and 816 downregulated genes after treatment with DPC-Exo^miR−218−5p^ (Fig. 5A, Table S6). The relative mRNA expression of six DEGs (*MSTN*, *PLS1*, *SFRP2*, *STAT1*, *TAP1*, and *TSR1*) was examined. RT-qPCR results were consistent with the sequencing data (Fig. 5B). GO and KEGG enrichment analyses of DEGs were conducted. The results showed enrichment of GO terms, including cellular process, biological regulation, cellular anatomical entity, and binding (Fig. 5C). HF growth and development-related GO terms, such as hair follicle development (GO:0001942), hair follicle morphogenesis (GO:0031,069), and hair follicle placode formation (GO:0060,789), were enriched. HF growth-related DEGs *KRT17*, *FOXE1*, and *EDAR* were upregulated in the DPC-Exo^miR−218−5p^ group. KEGG pathways associated with DEGs included the NOD-like receptor signaling pathway, TNF signaling pathway, and cytokine-cytokine receptor interaction (Fig. 5D). HF growth and development-related signaling pathways, such as Wnt/β-catenin, MAPK, TGF-β, PI3K-Akt, and JAK-STAT signaling, were identified. To further reveal the regulatory mechanism of DPC-Exo^miR−218−5p^ in HFSCs, common genes were selected between miR-218–5p predicted target genes and downregulated DEGs in the DPC-Exo^miR−218−5p^ group. The results identified 46 common genes, including HF development-related genes such as *SFRP2*, *LGR5*, and *GREM1* (Fig. 5E). Our previous study confirmed that miR-218–5p directly targeted SFRP2 in skin and HF development through the Wnt/β-catenin pathway [44]. Therefore, these results suggested that exosomal miR-218–5p played a pivotal role in HF growth and development by regulating transcription levels in HFSCs.

### 3.5 Exosomal miR-218–5p regulated HF growth in HF organ culture

**Fig. 6:**
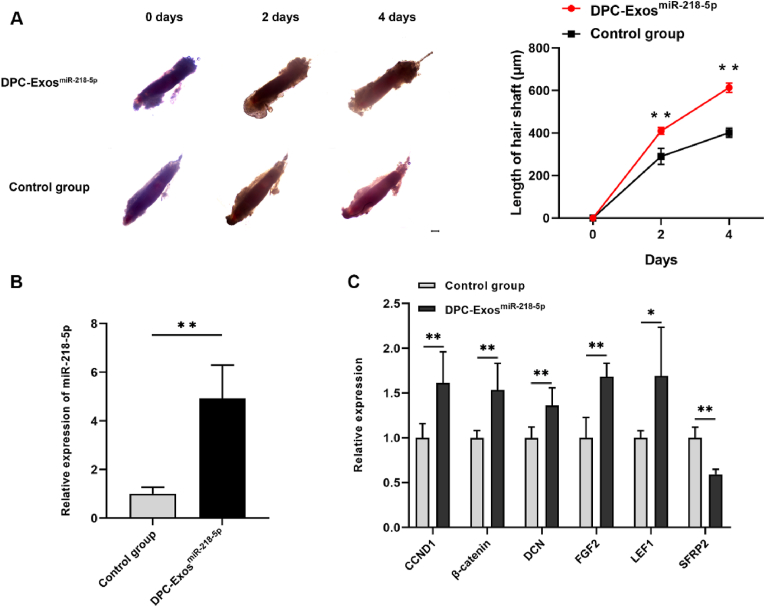
**DPC-Exos containing miR-218-5p promoted HF growth.** (A) HF morphology and hair shaft length in HF organ culture after treatment with DPC-Exos^miR^^−218−5p^ (unpaired two-tailed *t*-test, n = 3). (B) MiR-218–5p expression in HF organs treated with DPC-Exos^miR^^−218−5p^ (unpaired two-tailed *t*-test, n = 3). (C) Expression of HF development-related genes in HF organs treated with DPC-Exo^miR−218−5p^ (unpaired two-tailed *t*-test, n = 3). Scale bar = 200 μm ∗*P* < 0.05, ∗∗*P* < 0.01.

The role of exosomal miR-218–5p in regulating HF growth was examined using an *in vitro* Angora rabbit HF organ culture system. With exosomal miR-218–5p treatment, hair shaft length significantly increased compared with the control group from 0 to 4 days (*P* < 0.01, Fig. 6A). Expression of miR-218–5p was significantly upregulated in the HF organ (*P* < 0.01, Fig. 6B). After collecting HFs, the expression levels of HF growth and development-related genes were measured. The results showed that treatment with DPC-Exo^miR−218−5p^ significantly upregulated *β-catenin*, *CCND1*, *DCN*, *FGF2*, and *LEF1* mRNA expression, while significantly downregulating *SFRP2* mRNA expression *in vitro* (*P* < 0.05, Fig. 6C). These results demonstrated that exosomal miR-218–5p promoted HF growth *in vitro*.

## 4 Discussion

Exosomes are nanosized extracellular vesicles secreted by cells that play critical roles in intercellular communication, regulation of biological functions, and pathological processes, and have potential applications as diagnostic tools and therapeutic agents [45,46]. In previous studies, exosomes derived from DPCs regulated HF development and regrowth through cell–cell communication. DPC-Exos modulated HF growth-related genes in HFSCs, promoted HFSC proliferation, and inhibited HFSC apoptosis [[47], [48], [49]]. Low-passage DPCs effectively induce hair growth, whereas high-passage DPCs do not and generate only subepidermal HFs without dermal sheath encapsulation [50]. In this study, low-passage DPC-Exos promoted HFSC proliferation and inhibited HFSC apoptosis, thereby regulating HF growth and cyclic regeneration. This result highlights the importance of DPC passage in maintaining exosomal functional activity, which could promote cell proliferation and inhibit cell apoptosis in HFSCs.

MiRNAs, as key components of exosomal cargo, serve as disease biomarkers and therapeutic targe [51,52]. Several exosomal miRNAs have been reported to play essential roles in HF growth and development. For instance, miR-22–5p negatively regulates HFSC proliferation by targeting LEF1 [25], and miR-181a-5p promotes hair shaft growth by targeting WIF1 [26]. Compared with these miRNAs, miR-218–5p targets SFRP2, a different inhibitor of the Wnt/β-catenin pathway. These findings suggest that exosomal miRNAs can regulate HF growth through distinct target genes in the same signaling pathway, indicating a synergistic regulatory network. Using miRNA sequencing, we identified 36 DE miRNAs between low-passage and high-passage DPC-Exos, with miR-218–5p significantly upregulated in low-passage DPC-Exos. Through DiI labeling, siDrosha interference, and GW4869 inhibition, we confirmed that miR-218–5p is transported from DPCs to HFSCs via exosomes. RNA sequencing data further revealed 1947 DEGs in HFSCs treated with exosomal miR-218–5p, including 46 overlapping genes between predicted target genes and downregulated DEGs. All of them are related to HF growth and development, including *SFRP2* [53], *LGR5* [54], and *GREM1* [55]. These findings expand the regulatory mechanism of miR-218–5p beyond the previously reported SFRP2-Wnt/β-catenin axis, indicating involvement of multiple HF-related pathways, such as Wnt/β-catenin [56], MAPK [57], PI3K-Akt [58], and JAK-STAT signaling pathway [59]. In HF organ culture, exosomal miR-218–5p promoted hair shaft growth and regulated the expression of HF-related genes, providing evidence from a non-rodent model (rabbit) that supplements previous mouse studies [27]. This is particularly relevant for improving wool production in mammals, as rabbit models are closer to livestock species than mice. However, only *in vitro* experiments were conducted, and future *in vivo* studies are needed to validate the therapeutic potential of exosomal miR-218–5p. Additionally, the synergistic effects of other DE-miRNAs, such as miR-140–3p and miR-455–5p, were not explored and could be a focus of future research. Future work could also focus on engineering exosomes to enhance miR-218–5p delivery efficiency, thereby providing new therapeutic strategies for hair loss treatment and wool production.

## 5 Conclusion

**Fig. 7:**
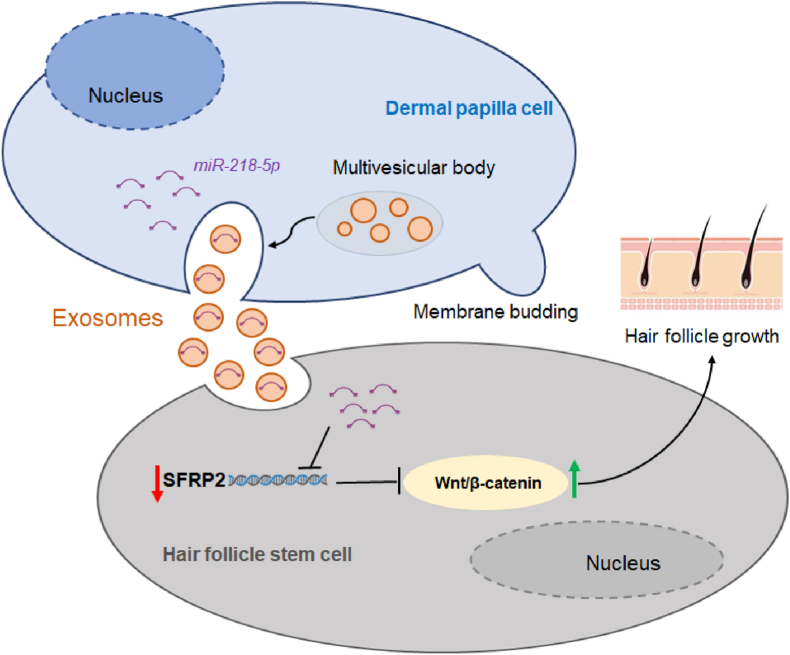
A schematic showing exosomal miR-218–5p derived from DPCs positively regulating HF growth by targeting SFRP2.

This study revealed that low-passage DPC-Exos enhances HFSC proliferation. Specifically, miR-218–5p derived from DPC-Exos dysregulated HF growth- and development-related gene expression in HFSCs and promoted hair shaft growth *in vitro*. Therefore, exosomal miR-218–5p could serve as a novel therapeutic target for hair-related diseases and a potential biomarker for improving wool production in mammals (Fig. 7).

## CRediT authorship contribution statement

**Bohao Zhao:** Writing – original draft, Methodology, Conceptualization. **Jiawei Cai:** Methodology, Investigation. **Miaocheng Wang:** Software, Methodology. **Jiali Li:** Methodology, Investigation. **Shuyu Yao:** Validation, Methodology. **Zhiyuan Bao:** Software. **Yang Chen:** Writing – review & editing. **Xinsheng Wu:** Writing – review & editing, Supervision, Conceptualization.

## Availability of data and materials

All datasets used and analyzed during the current study are available from the corresponding author on reasonable request.

## Ethics approval and consent to participate

All animal experimental procedures were approved by the Animal Care and Use Committee of Yangzhou University (Approval No.: 202103358).

## Funding

This research was funded by the China Agriculture Research System of MOF and MARA (CARS-43-A-1), National Natural Science Foundation of China (Grant No. 32102529), Natural Science Foundation of Jiangsu Province (BK20231332).

## Declaration of competing interest

The authors declare that they have no known competing financial interests or personal relationships that could have appeared to influence the work reported in this paper.

## Appendix A Supplementary data

Table S1. siRNA sequences used in this study.Multimedia component 1

Table S2. The quality control and mapping data from miRNA sequencing.Multimedia component 2

Table S3. The quality control and mapping data from RNA sequencing.Multimedia component 3

Table S4. Primers for qPCR with reverse transcription (RT-qPCR) analysis.Multimedia component 4

Table S5. Differentially expressed mRNAs between DPC-Exos P1 and DPC-Exos P8.Multimedia component 5

Table S6. Differentially expressed genes between the DPC-Exo^miR−218−5p^ group and the control group.Multimedia component 6

Additional file 1. Origin blot for Western blot and Wes analysis.Multimedia component 7
