# Supplementary material for: Exosomal miRNA-218–5p derived from low-passage dermal papilla cells modulates hair follicle growth and development
Source: Noncoding RNA Res. 2026 Jan 15;18:1–11. doi: 10.1016/j.ncrna.2026.01.004 (PMC12830253; doi:10.1016/j.ncrna.2026.01.004)
Supplement: Supplementary file 7 — Additional file 1. Origin blot for Western blot and Wes analysis.Multimedia component 7 [file mmc7.pdf]

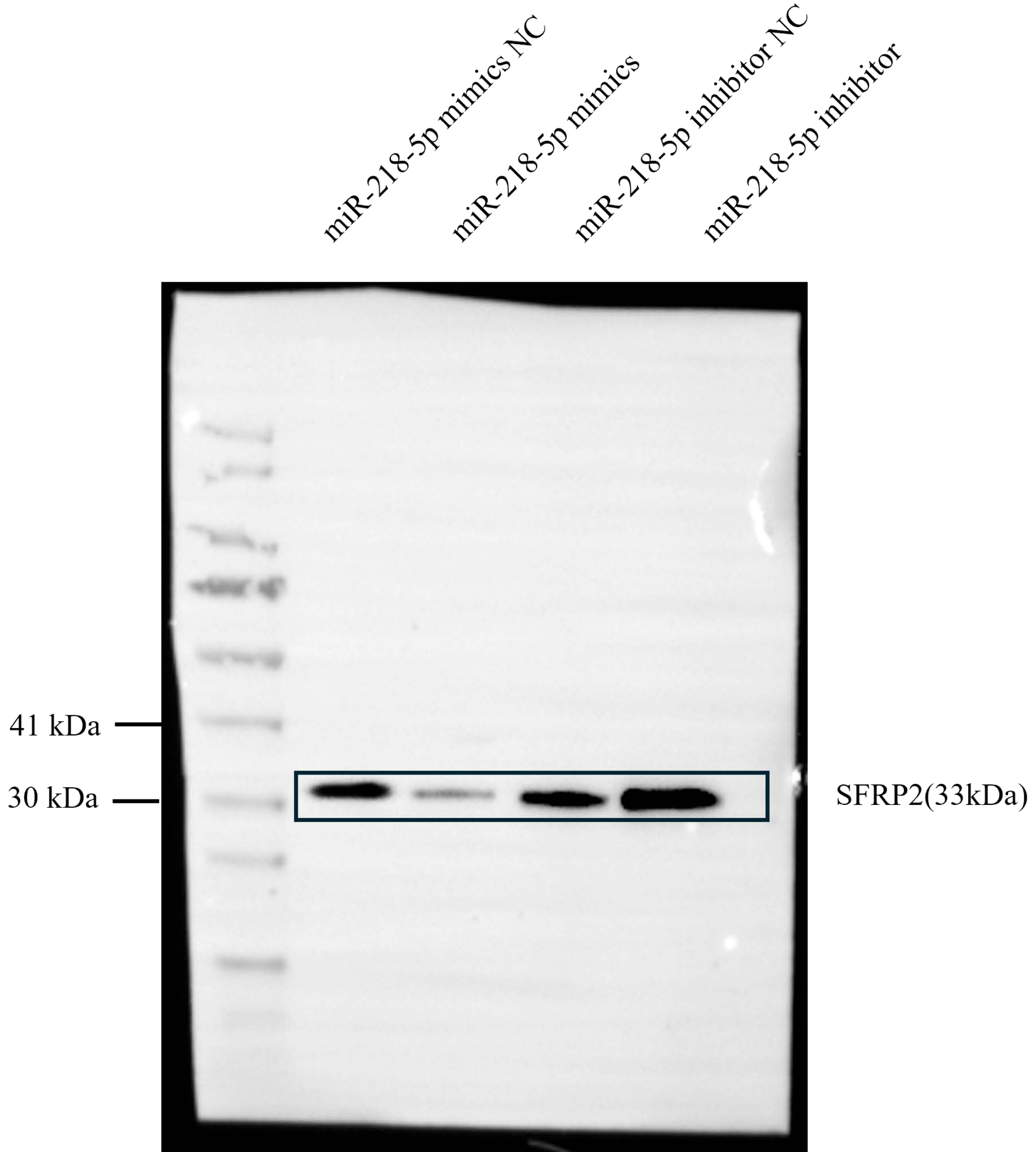
miR-218-5p mimics NC
miR-218-5p mimics
miR-218-5p inhibitor NC
miR-218-5p inhibitor
41 kDa
30 kDa
SFRP2(33kDa)

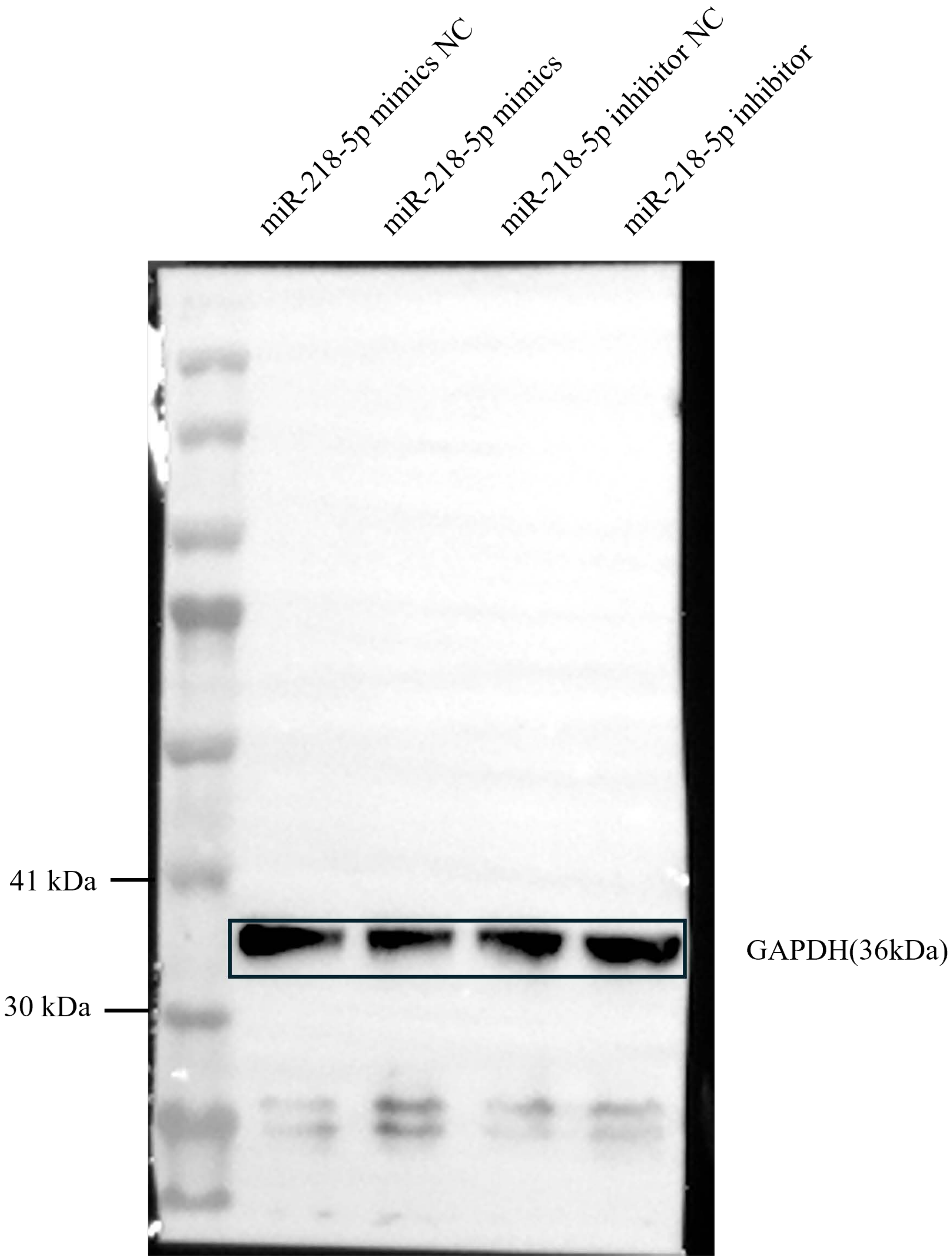
miR-218-5p mimics NC
miR-218-5p mimics
miR-218-5p inhibitor NC
miR-218-5p inhibitor
41 kDa
30 kDa
GAPDH(36kDa)

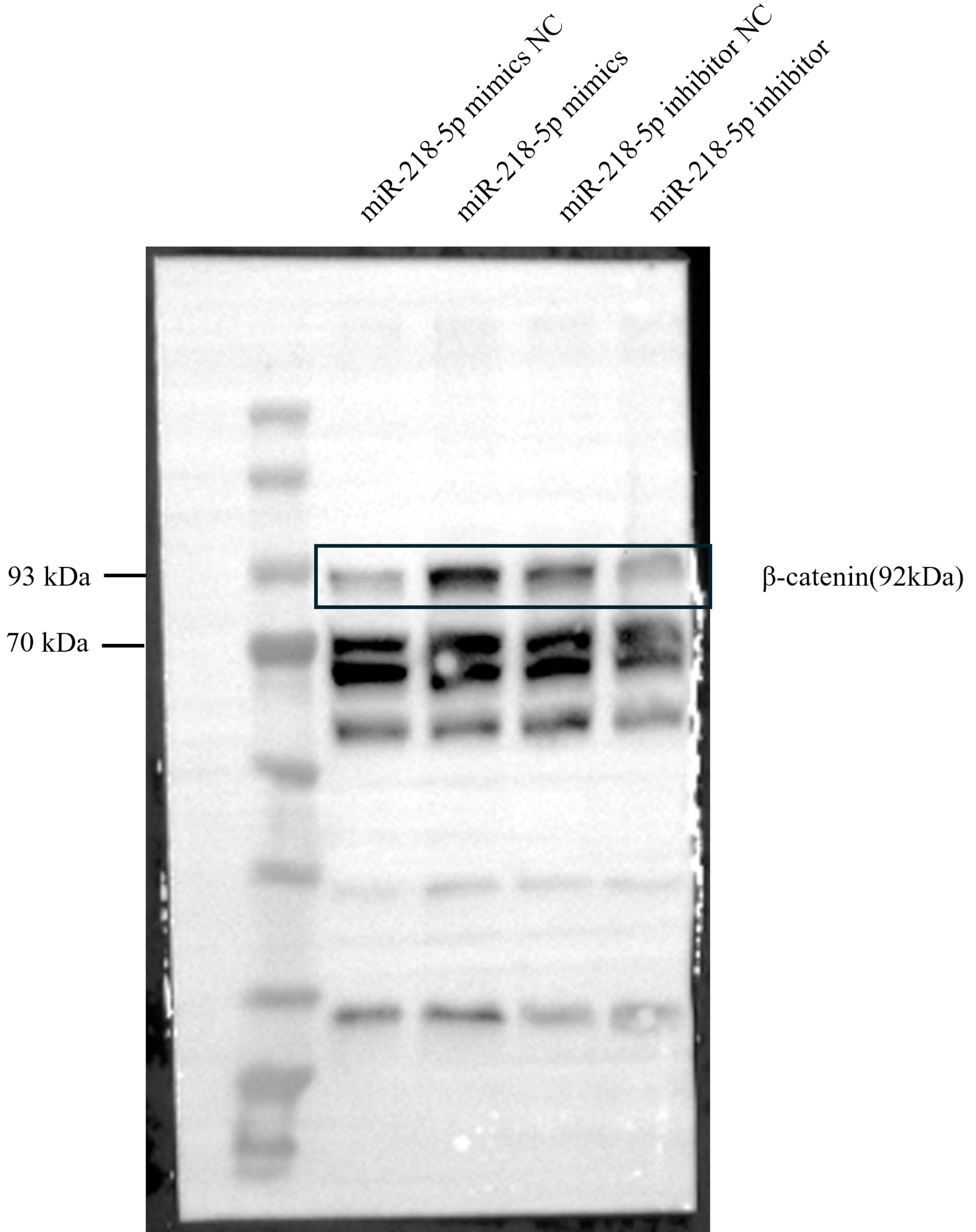
miR-218-5p mimics NC
miR-218-5p mimics
miR-218-5p inhibitor NC
miR-218-5p inhibitor
93 kDa
70 kDa
β-catenin(92kDa)

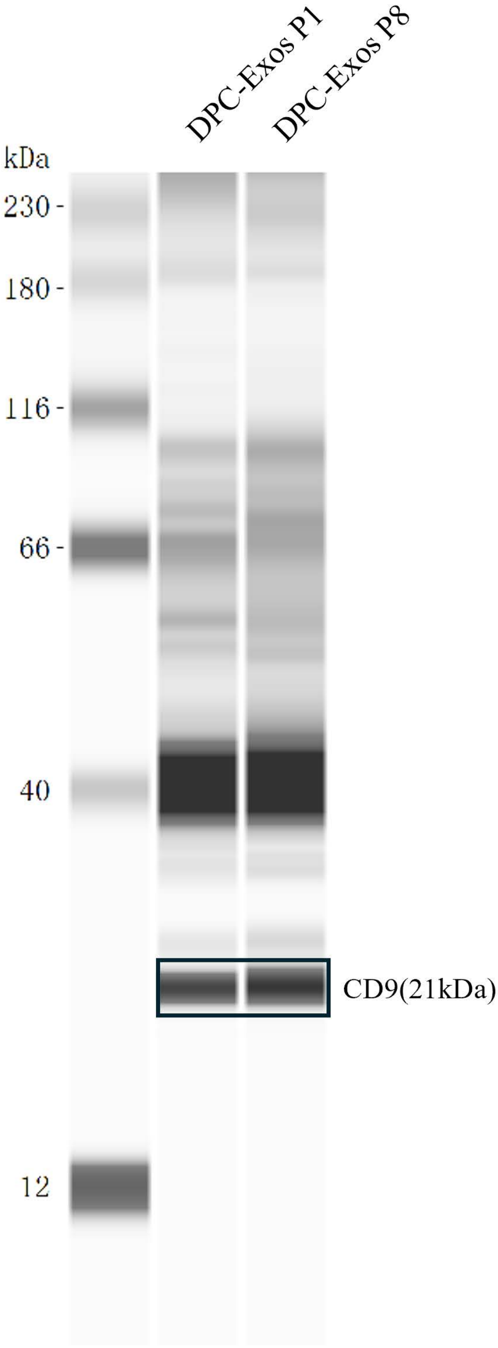
DPC-Exos P1
DPC-Exos P8
kDa
230
180
116
66
40
12
CD9(21kDa)

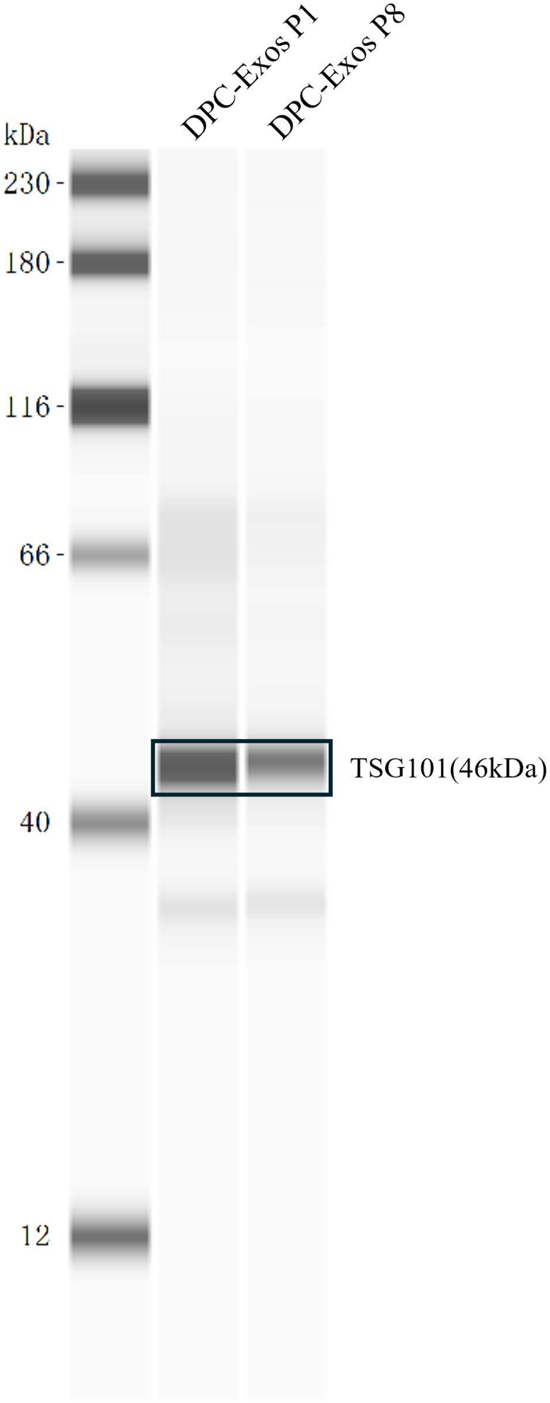

DPC-Exos P1
DPC-Exos P8
kDa
230
180
116
66
40
12
TSG101(46kDa)

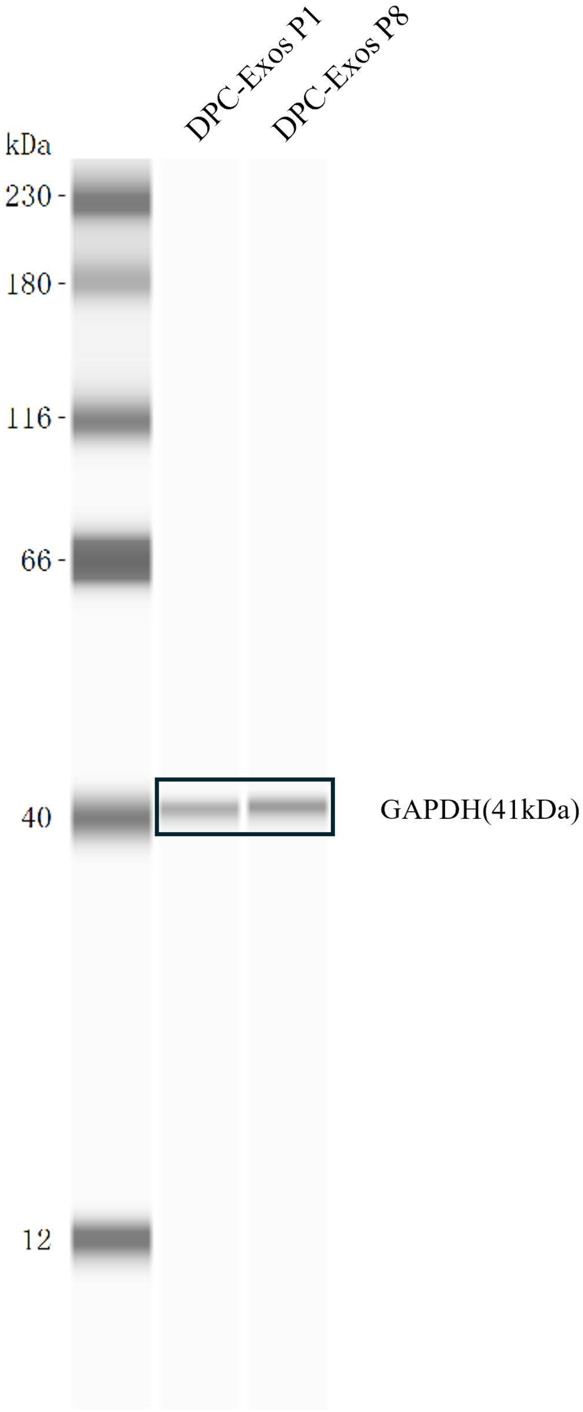
DPC-Exos P1
DPC-Exos P8
kDa
230
180
116
66
40
12
GAPDH(41kDa)

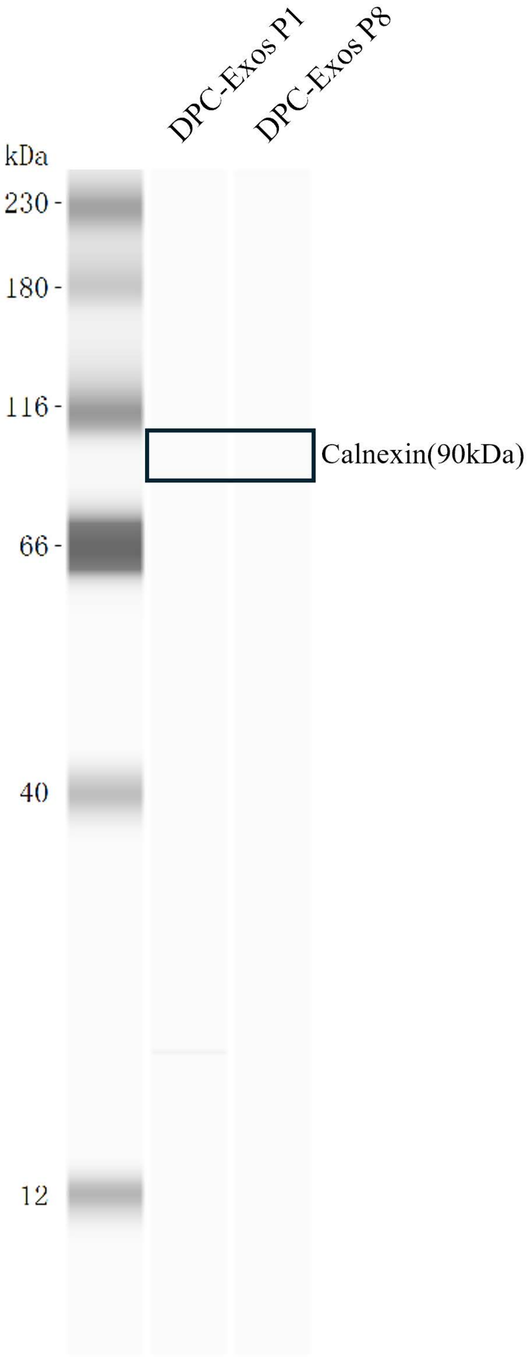

DPC-Exos P1
DPC-Exos P8
kDa
230
180
116
66
40
12
Calnexin(90kDa)
